# Tattooing and piercing are associated with symptoms of ADHD: A cross-sectional study of non-clinical adults

**DOI:** 10.1192/j.eurpsy.2022.255

**Published:** 2022-09-01

**Authors:** M. Glans, S. Bejerot, J. Dossou Nilsson

**Affiliations:** 1 Faculty of Medicine and Health, University Health Care Research Centre, Örebro, Sweden; 2 Örebro University, Faculty Of Medicine And Health, Örebro, Sweden

**Keywords:** body piercing, Impulsivity, Attention Deficit Disorder with Hyperactivity, tattoing

## Abstract

**Introduction:**

Previous studies suggest that individuals with tattoos and piercings exhibit higher impulsive personality traits compared to peers without body modifications. No studies on body modifications and core-symptoms of ADHD are available.

**Objectives:**

This study aimed to compare self-reported ADHD symptoms between non-clinical adults with and without body modifications.

**Methods:**

A non-clinical adult Swedish population (n=815) completed the Adult ADHD self-report scale (ASRS-v1.1) and answered questions concerning body modification. ADHD diagnosis served as exclusion criterion. Three grouping variables were analysed separately; tattoo status, piercing status and a combination of having both tattoo and piercing. Linear regression compared mean ASRS total- and subscale scores between individuals with and without body modification according to each grouping variable, while adjusting for candidate covariates age and sex.

**Results:**

The prevalence of each body modification variable was; 30% for tattoo, 18% for piercing other than earlobe and 12% for combination of tattoo and piercing. Any combination of body modification was associated with significantly higher ASRS total- and subscale scores compared to no body modification. The most pronounced differences between groups were for the combination of tattoo and piercing, and on the hyperactivity/impulsivity (HI) subscale; revealing adjusted mean differences of 4.3 points (range 0-72) on the ASRS-total score (p <0.001) and 2.6 points (range 0-36) on the ASRS HI subscale (p <0.001).

**Conclusions:**

Body modification was associated with more pronounced ADHD core symptoms amongst non-clinical adults. Although statistically significant, the clinical significance is uncertain. The prevalence rates of body modifications in our cohort indicate that they are becoming cultural normal.

**Disclosure:**

No significant relationships.

